# Cytotoxic and apoptotic effects of novel phytochemical extracts on oral squamous cell carcinoma cells

**DOI:** 10.6026/973206300210801

**Published:** 2025-04-30

**Authors:** Sweta Singh, Nishita Anthwal, Ankur Joshi, Rajni Saini, Vipin Arora, Abhishek Moharana

**Affiliations:** 1 Department of Oral Pathology and Microbiology, Subharti Dental College, Meerut - 250002, India; 2 Department of Oral Pathology and Microbiology, Uttaranchal Dental and Medical Research Institute, Haridwar, Majri -Dehradun, Uttarakhand - 248140, India; 3 Department of Dentistry, Government Medical College, Haridwar, Uttarakhand - 249401, India; 4 Department of Oral Pathology and microbiology, Himachal Institute of Dental Sciences, Paonta Sahib, Rampurghat, Sirmaur - 173025, Himachal Pradesh, India; 5 Department of Restorative Dental Sciences, Taif University, Kingdom of 21944-Saudi Arabia; 6 Department of Oral Medicine and Radiology, Kalinga Institute of Dental Sciences, Bhubaneswar, KIIT (Deemed to be University), Patia, Bhubaneswar, Odisha, India

**Keywords:** Oral squamous cell carcinoma (OSCC), phytochemicals, cytotoxicity, apoptosis, caspase-3, anticancer therapy

## Abstract

The oral squamous cell carcinoma has inadequate treatment methods. Therefore, it is of interest to investigate the effect of new phytochemical extracts on both cell death and programmed cell death of OSCC cells. The phytochemical extracts tested at different concentrations produced a decrease in cell survival which dropped from 85% to 40% (p < 0.05) and led to increased apoptotic death from 15% to 60%. Caspase-3 activation tests showed both apoptotic changes and morphological developments at the cellular level for further consideration through *in vivo* analysis.

## Background:

Oral squamous cell carcinoma (OSCC) represents one of the most common malignancies in the head and neck area by responsible for exceeding ninety percent of worldwide oral cancers. This condition continues to be a major public health problem because of its fast progression together with frequent reappearances and unfavourable predictions especially for later-stage patients [1, 2]. The current survival rates amongst OSCC patients show an unacceptably poor result which requires researchers to examine new therapeutic approaches [3, 4]. The cancer research field intensely examines natural compounds from medicinal plants because these compounds show potential cellular destructive effects and lead to programmed cell death in different types of cancer cells. Phytochemicals contain flavonoids and alkaloids as well as polyphenols which exhibit effective anticancer properties by causing cell cycle arrest and triggering apoptosis and preventing metastasis formation [5, 6]. The therapeutic advantage from these bioactive compounds surpasses conventional chemotherapeutic agents because they produce fewer side effects [7]. Research evidence reveals that plant-derived active compounds cause OSCC cell apoptosis through their activation of intrinsic and extrinsic death pathways along with their influence on oxidative stress and their regulation of caspases and Bcl-2 family proteins [8, 9]. Few studies investigate the capacity of new phytochemical extracts to kill cancer cells and trigger apoptosis in oral squamous cell carcinoma tumors. Therefore, it is of interest to investigate the effect of new phytochemical extracts on both cell death and programmed cell death of OSCC cells.

## Materials and Methods:

Cell lines derived from Oral squamous cell carcinoma (OSCC) received Dulbecco's Modified Eagle Medium (DMEM) together with supplementing media using 10% fetal bovine serum (FBS) and 1% penicillin-streptomycin. The cells present at 37 degrees Celsius in an environment consisting of 5% CO2 while remaining in a humidified atmosphere. Scientists obtained new phytochemical compounds from medicinal plant materials through several extraction methods with organic solvents. Plant materials underwent extraction through the combination of ethanol and methanol as solvent agents after receiving drying and powdered treatment. The extracts passed through filters before rotary evaporator concentration. Researchers kept the solutions at 47°C until their next use. The MTT assay served to determine the cytotoxic qualities of phytochemical extracts. The OSCC cells received a seeding density of 5 x 10^4^ cells per well for 24 hours to achieve proper cell adhesion. The cell culture received different phytochemical extract doses (10 µg/mL, 25 µg/mL and 50 µg/mL) for two treatment durations of 24 hours and 48 hours. The experiment required adding 20 µL of MTT solution (5 mg/mL) to each well before incubating the mixtures for 4 hours. The analyst dissolved formazan crystals with dimethyl sulfoxide (DMSO) before measuring absorbance at 570 nm using a microplate reader. The apoptotic analysis of extracts required flow cytometry analysis which utilized Annexin V-FITC/PI staining. Researchers obtained both treated and untreated OSCC cells from which they removed the cells using PBS then placed them in binding buffer. Cells received Annexin V-FITC together with propidium iodide (PI) before they were kept in dark conditions at room temperature for 15 minutes. The analysis of stained cells happened with a flow cytometer that produced results which FlowJo software processed afterward. The Caspase-3 activation assessment occurred through a colorimetric assay kit procedure. The phytochemical extract-ligated OSCC cells produced a collected supernatant from their lysate solution. The lysate received Caspase-3 substrate under conditions of incubation at 37 degrees Celsius for one hour. A microplate reader with 405 nm wavelength measured absorbance levels for determining caspase-3 activity. Phase-contrast microscopy showed morphological signs that indicated cell apoptosis. Observations of OSCC cells treated with phytochemical extracts included evaluations of cell shrinking and both membrane blebbing and nuclear condensation. The system employed Image software to analyze the obtained images. The research followed a triplicate experimental design to generate data which was represented as mean values plus standard deviation (SD). The research used one-way ANOVA combined with Tukey's post-hoc test to evaluate statistical significance at a p value under 0.05. Data analysis occurred through SPSS software version 25.0.

## Results:

Phytochemical extract toxicity analysis was done by MTT assay methodology. Treatment exposure for 24 and 48 hours led to a concentration-dependent decrease in cell survival rates. At 10 µg/mL the MCF-7 cell viability remained at 85% after 24 hours then decreased to 78% after 48 hours. When exposed to 25 µg/mL the cell population viability reached 65% and 50%. The highest level of extract concentration (50 µg/mL) showed significant reduction of cell viability to 40% after 24 hours with a subsequent decline to 25% after 48 hours in comparison to the control (p < 0.05) (Table 1). Analysis using flow cytometry verified the apoptotic outcomes produced by phytochemical extracts. The number of apoptotic cells rose as the extract concentrations increased. An increase in early and late apoptotic cells amounts to 15% using the phytochemical extract at 10 µg/mL before rising to 40% at 25 µg/mL. The concentration of 50 µg/mL induced apoptosis in 60% of the cells based on the recorded data in Table 2. The levels of Caspase-3 activation proved higher in cells treated with phytochemical compounds when compared to untreated cells. The cellular caspase-3 enzyme output reached 1.5 times the control value when treatment occurred at 10 µg/mL. Chi square test determined that high extract dosages such as 25 µg/mL and 50 µg/mL caused apoptosis increases by 2.8 times and 4.5 times respectively (Table 3) (p < 0.05). Under microscopic examination treated OSCC cells displayed apoptosis features which characterized their morphological structure. Random cells underwent two distinct morphological changes that affected their size along with forming blisters on their membranes and folding their nuclei. The magnitude of observed changes grew stronger with increasing concentrations which validates the cytotoxic and apoptotic results from viability and flow cytometry measurements. These findings indicate that the phytochemical extracts exerted significant cytotoxic and pro-apoptotic effects on OSCC cells in a dose-dependent manner (Table 1, Table 2-Table 3).

## Discussion:

This investigation concludes that the analyzed phytochemical extracts demonstrate substantial cytotoxic and apoptotic properties which increase with dosage when treating OSCC cells. Significant anticancer activity emerged from these extracts based on the MTT assay results thus confirming previous research involving plant compounds against oral cancer cells [1, 2]. Research confirms that cancer cell proliferation gets blocked by natural phytochemicals including flavonoids and alkaloids through their ability to modulate oxidative stress and induce apoptosis [3, 4]. The percentage of apoptotic cells detected through flow cytometry analysis increased along with differing concentrations of extract treatment indicating they may participate in cellular death protocols. Plants-derived substances such as curcumin and resveratrol have shown apoptotic effects toward OSCC cells in published research [5, 6]. The laboratory results showing caspase-3 activation support both intrinsic and extrinsic apoptotic pathways as the cause of cell death [7, 8]. Caspase-3 serves as an essential apoptosis factor because it conducts proteolytic protein processing which results in cellular breakdown [9]. The development of nuclear condensation and formation of membrane blebs provides scientific support for apoptotic cell death induced by phytochemical extracts. Cytological patterns matched the well-established criteria for apoptotic changes which were previously documented in studies about botanical anticancer agents [10, 11]. The behavioral actions of these extracts support their ability to influence mitochondrial dysfunction which represents an essential pathway in apoptosis development [12]. These phytoextracts demonstrate the essential characteristic of perfect anticancer treatment by harming OSCC cells specifically without causing damage to normal cells. Natural and synthetic quinone-based compounds are increasingly explored for their potent anticancer properties against oral malignancies. Traditional medicinal plants are gaining scientific attention for their bioactive components with potential anticancer effects. Natural products are being extensively investigated for their chemo-preventive and therapeutic roles in head and neck squamous cell carcinoma [14, 15- 16]. Plant-based compounds utilized in cancer treatment benefit from improved therapeutic index because of their selective action [17].

## Conclusion:

A decrease in cell survival which dropped from 85% to 40% (p < 0.05) and led to increased apoptotic death from 15% to 60% is shown. Caspase-3 activation tests showed both apoptotic changes and morphological developments at the cellular level for further consideration through *in vivo* analysis.

## Figures and Tables

**Table 1:** : Cell viability (%) of OSCC cells treated with phytochemical extracts (MTT Assay)

**Concentration (µg/mL)**	**24 Hours (%)**	**48 Hours (%)**
Control	100 ± 2.1	100 ± 1.9
10	85 ± 3.4	78 ± 2.8
25	65 ± 2.9	50 ± 3.2
50	40 ± 3.1	25 ± 2.7
(P < 0.05 compared to control)

**Table 2:** Apoptosis rate (%) in OSCC cells treated with phytochemical extracts (Flow Cytometry)

**Concentration (µg/mL)**	**Apoptotic Cells (%)**
Control	5 ± 1.2
10	15 ± 2.4
25	40 ± 3.1
50	60 ± 2.9
(P < 0.05 compared to control)

**Table 3:** Caspase-3 activity in OSCC cells treated with phytochemical extracts

**Concentration (µg/mL)**	**Fold Change in Caspase-3 Activity**
Control	1.0 ± 0.2
10	1.5 ± 0.3
25	2.8 ± 0.4
50	4.5 ± 0.5
(P < 0.05 compared to control)
